# Factors related to subjective satisfaction following microendoscopic foraminotomy for cervical radiculopathy

**DOI:** 10.1186/s12891-018-1947-4

**Published:** 2018-01-24

**Authors:** Juichi Tonosu, Hirohiko Inanami, Hiroyuki Oka, Yuichi Takano, Hisashi Koga, Yohei Yuzawa, Ryutaro Shiboi, Yasushi Oshima, Satoshi Baba, Sakae Tanaka, Ko Matsudaira

**Affiliations:** 1 Department of Orthopedic Surgery, Kanto Rosai Hospital, 1-1 Kizukisumiyoshicho, Nakahara-ku, Kawasaki-city, Kanagawa 211-8510 Japan; 2 Department of Orthopedic Surgery, Inanami Spine and Joint Hospital, 3-17-5 Higashishinagawa, Shinagawa-ku, Tokyo, 140-0002 Japan; 3 Department of Orthopedic Surgery, Iwai Orthopaedic Medical Hospital, 8-17-2 Minamikoiwa, Edogawa-ku, Tokyo, 133-0056 Japan; 4 Department of Medical Research and Management for Musculoskeletal Pain, 22nd Century Medical and Research Center, Faculty of Medicine, The University of Tokyo, 7-3-1 Hongo Bunkyo-ku, Tokyo, 113-8655 Japan; 5 Department of Orthopedic Surgery, Faculty of Medicine, The University of Tokyo, 7-3-1 Hongo Bunkyo-ku, Tokyo, 113-8655 Japan

**Keywords:** Cervical radiculopathy, Microendoscopic foraminotomy, Satisfaction

## Abstract

**Background:**

Microendoscopic foraminotomy has been reported to be effective for the treatment of cervical radiculopathy, using outcome measurement scores such as the neck disability index (NDI) and numerical rating scale (NRS). However, the scores for spine surgery do not always reflect the true subjective satisfaction of the patient. The purpose of this study was to evaluate factors related to subjective satisfaction following microendoscopic foraminotomy for cervical radiculopathy.

**Methods:**

The subjects consisted of consecutive patients who underwent microendoscopic foraminotomy for cervical radiculopathy. Patient background information and operative data were collected. The NDI, the NRS score for the neck, upper back, and arm, and the EuroQOL-5D (EQ-5D) were assessed preoperatively and 1 year postoperatively. Postoperative subjective satisfaction was also assessed as a direct evaluation of satisfaction, and willingness to undergo the same operation if needed was assessed as an indirect evaluation.

**Results:**

A total of 42 patients were included in this study. The mean age was 52.9 ± 11.8 years; 19.0% were female and 81.0% were male. The operation time for one level was 57.7 min and the estimated blood loss was minimal in most cases. All NDI, NRS, and EQ-5D scores improved significantly postoperatively. Univariate analyses revealed that the factors related to subjective satisfaction were younger age, non-smoking status, high preoperative NDI score, and low postoperative NRS score for the arm. Factors related to the willingness to undergo the same operation if needed were high preoperative NDI scores, high preoperative NRS scores for the arm, and low preoperative EQ-5D scores.

**Conclusions:**

Factors related to subjective satisfaction following microendoscopic foraminotomy include younger age, non-smoking status, high preoperative NDI score, high preoperative NRS score for the arm, low preoperative EQ-5D score, and a low postoperative NRS score for the arm.

## Background

Radiating pain to the arm caused by cervical radiculopathy (CR) is a common cause of disability in activities of daily life. CR is a clinical condition resulting from the compression of cervical nerve roots. Compression of the nerve root may occur by impingement due to disc herniation or bony osteophytes. It typically presents as unilateral arm pain and upper back pain around the scapula, and sometimes, motor weakness of the arm [1, 2]. Randhakrishnan et al. estimated that the annual incidence of cervical radiculopathy is 107.3 per 100,000 for men and 63.5 per 100,000 for women [3]. Some studies have suggested that the natural history of CR is favorable and self-limiting [3, 4], and nearly 90% of patients have symptomatic improvement with conservative treatment [5, 6]. However, some patients with CR do not respond to conservative therapy and subsequently require surgery.

Surgical methods include anterior cervical decompression and fusion (ACDF) or disc replacement, and foraminotomy using a posterior approach. Both methods have been reported to be effective for the treatment of CR [1, 2, 7]. Posterior foraminotomy is performed by an open approach or minimally invasive approach using a microscope or endoscope. A review of the literature has revealed that foraminotomy for CR provides good clinical results by both the open and minimally invasive approach, although the minimally invasive approach results in less blood loss, shorter operation time, and shorter hospital stays than the open approach [2, 8–10]. However, outcome measurement scores for spine surgery, such as the neck disability index (NDI), do not always reflect the true subjective satisfaction of the patient [11, 12]. True subjective satisfaction following surgery has not been well-estimated.

Since the introduction of microendoscopic foraminotomy in 2002 [13], we have performed microendoscopic foraminotomy for patients with severe CR. The purpose of this study was to evaluate factors related to true subjective satisfaction following microendoscopic foraminotomy for CR.

## Methods

### Subjects

We typically diagnose CR using physical findings, X-rays, magnetic resonance imaging, and computed tomography. In addition, we sometimes perform electromyography and transforaminal epidural nerve block injections during diagnosis. Patients who do not respond sufficiently to a three-month course of conservative therapy proceed to surgery. We collected the data of consecutive patients who underwent microendoscopic foraminotomy between May 2012 and June 2014 from the operation database of our hospital. We performed microendoscopic foraminotomy on 44 patients in this time frame. Of these 44 patients, we excluded two who did not fully complete the preoperative and postoperative evaluations. Therefore, a total of 42 subjects were included in this study.

Patient background information was collected, including age, sex, height, weight, and smoking status. Body mass index (BMI) was calculated from the height and weight data. The number and level of the operated foramen, operated side, operation time, estimated blood loss, complications related to the operation, and occurrence of re-operation in 1 year were all obtained from medical records. We also obtained the following outcome scores by self-written questionnaire. The NDI score [14] was assessed preoperatively and 1 year postoperatively to evaluate the degree of disability. The numerical rating scale (NRS) score for the neck, upper back, and arm was also assessed to evaluate the degree of pain. The area of pain was shown diagrammatically on the questionnaire. The EuroQOL-5D (EQ-5D) score was also used to evaluate disability by assessing comprehensive quality of life [15]. We used the EQ-5D-3 L version and calculated the score according to the previous literature [16]. Postoperative subjective satisfaction and willingness to undergo the same operation if needed were also assessed, using a seven-level rating scale with the following levels: extremely satisfied/willing, very satisfied/willing, satisfied/willing, borderline, unsatisfied/unwilling, very unsatisfied/unwilling, and extremely unsatisfied/unwilling. We regarded the former question (subjective satisfaction) as a direct evaluation of satisfaction and the latter question (willingness to undergo the same operation if needed) as an indirect evaluation of satisfaction. We divided the patients into a satisfied and unsatisfied group and a willing and unwilling group so that we could evaluate factors related to true subjective satisfaction following microendoscopic foraminotomy for CR.

### Statistical methods

We compared the baseline characteristics of both groups, and analyzed the factors related to subjective satisfaction and the willingness to undergo the same operation if needed. To evaluate the relationship between the response to the subjective satisfaction survey and willingness to undergo the same operation if needed, a correlation analysis between the response to the subjective satisfaction survey and the willingness to undergo the same operation was evaluated by Fisher’s exact test. Descriptive statistics are presented as means and standard deviations or frequencies and percentages. Between-group differences in baseline characteristics were evaluated using Fisher’s exact test for categorical variables and the Student’s t-test for continuous variables. In the satisfaction analysis, we converted the aforementioned seven-level rating scale into a two-level rating scale. We regarded the median answer of “borderline” as “unsatisfied” and “unwilling.” Subjects were grouped into a satisfied and unsatisfied group, and a willing and unwilling group based on their responses.

Statistical analysis was performed using the JMP 11.0 software program (SAS Institute, Cary, NC, USA). A *P* value < 0.05 was considered significant.

## Results

A total of 42 patients were included in this study. The mean age was 52.9 ± 11.8 years; 19.0% were female and 81.0% were male. The baseline characteristics and preoperative evaluations are shown in Table 1.

**Table 1:** Demographic data (*n* = 42)

Age	52.9 ± 11.8
Female	8 (19.0)
BMI (kg/m^2^)	24.2 ± 4.3
Smoking status	6 (14.3)
NDI (0–100)	32.8 ± 14.7
NRS; neck (0–10)	4.4 ± 3.2
NRS; upper back (0–10)	4.3 ± 3.5
NRS; arm (0–10)	5.4 ± 3.3
EQ-5D (0–1)	0.67 ± 0.13

Data are shown as mean ± SD or number of participants (%). BMI, body mass index; NDI, Neck disability index; NRS, numerical rating scale; EQ-5D, EuroQOL-5D

Details of the operations are shown in Table 2. One-level foraminotomy was performed in 29 cases and two-level foraminotomy in 13 cases. The operated side of the two-level foraminotomies was the same side in all 13 cases. The cervical level most frequently addressed was C5/6, followed by C6/7. Herniotomy was performed in addition to foraminotomy in seven cases. The estimated blood loss was minimal in almost all cases with the exception of two cases with blood loss of 100 ml and 500 ml, respectively. Much of the bleeding in these cases resulted from the venous plexus craniad to the nerve root. Three cases exhibited temporary postoperative muscle weakness, although they recovered in 1 month, 6 months, and 1 year, respectively. Additional herniotomy had been performed in one of these three cases. There were no incidences of dural tear or nerve root injury, and no cases of re-operation within 1 year.

**Table 2:** Details of the operations

Number of operated foramen	1	29
	2	13
Level of the operated foramen	C4/5	2
	C5/6	30
	C6/7	19
	C7/T1	4
Operated side of foramen (cases)	Right / Left	24 / 31 (18 / 24)
Operation time (min.)		72.7 ± 32.5
Operation time for one level (min.)		57.7 ± 27.1
Complications		
Temporary muscle weakness		3 (7.0)
Dural tear		0 (0.0)
Nerve root injury		0 (0.0)
Re-operation		0 (0.0)

Data below operation time are shown as mean ± SD or number of participants (%)

All NDI scores, NRS scores for the neck, upper back, and arm, and EQ-5D scores improved significantly postoperatively (Table 3). The number of patients who were subjectively satisfied with the operation was 34, whereas 8 were not. The number of patients who would be willing to undergo the same operation if needed was 36, whereas 6 would be unwilling. The responses to the subjective satisfaction survey and willingness to undergo the same operation were significantly related (*P* = 0.0079). Details of the subjective satisfaction survey and willingness to undergo the same operation if needed are shown in Table 4. Univariate analyses of factors related to subjective satisfaction are shown in Table 5. There were significant associations between subjective satisfaction at 12 months after the operation and age (*P* = 0.0062), smoking status (*P* = 0.0456), preoperative NDI score (*P* = 0.0209), and NRS score for the arm (*P* = 0.0424). There were significant associations between the willingness to undergo the same operation if needed and preoperative NDI score (*P* = 0.0109), NRS score for the arm (*P* = 0.0379), and EQ-5D score (*P* = 0.0140) (Table 6).

**Table 3:** Pre- and postoperative scores

	Pre-operation	Post-operation	*P*	Pre-operation minus post-operation
NDI (0–100)	32.8 ± 14.6	14.8 ± 11.6	< 0.0001*	18.0
NRS; neck (0–10)	4.4 ± 3.2	1.8 ± 2.3	< 0.0001*	2.6
NRS; upper back (0–10)	4.3 ± 3.5	1.5 ± 2.2	< 0.0001*	2.8
NRS; arm (0–10)	5.4 ± 3.3	2.0 ± 2.6	< 0.0001*	3.4
EQ-5D (0–1)	0.67 ± 0.13	0.81 ± 0.14	< 0.0001*	− 0.14

Data are shown as mean ± SD or number of participants (%). *: *P* < 0.05

NDI, Neck disability index; NRS, numerical rating scale; EQ-5D, EuroQOL-5D

**Table 4:** Details of subjective satisfaction and willingness to undergo the same operation if needed

	Subjective satisfaction	Willingness to undergo the same operation if needed
Extremely satisfied/willing	12	15
Very satisfied/willing	14	14
Satisfied/willing	8	7
Borderline	4	5
Unsatisfied/unwilling	3	0
Very unsatisfied/unwilling	1	1
Extremely unsatisfied/unwilling	0	0
Total	42	42

**Table 5:** Factors related to subjective satisfaction

		Satisfied (*n* = 34)	Unsatisfied (*n* = 8)	*P*
Age		50.5 ± 10.3	62.9 ± 3.8	0.0062*
Sex	Male (*n* = 34)	76.5	23.5	0.3163
	Female (*n* = 8)	100	0	
BMI (kg/m^2^)		24.6 ± 4.5	22.4 ± 2.7	0.1838
Smoking status	Yes	50.0	50.0	0.0456*
	No	91.3	8.7	
Number of operated foramen	1 (*n* = 29)	82.8	17.2	0.6861
	2 (*n* = 13)	76.9	23.5	
Operated side	Right (*n* = 18)	83.3	16.7	1.0000
	Left (*n* = 24)	79.2	20.8	
Pre-operation				
NDI (0–100)		35.4 ± 14.4	22.2 ± 10.6	0.0209*
NRS; neck (0–10)		4.4 ± 3.0	4.5 ± 4.2	0.9077
NRS; upper back (0–10)		4.1 ± 3.5	5.3 ± 3.7	0.4010
NRS; arm (0–10)		5.4 ± 3.3	5.8 ± 3.9	0.7661
EQ-5D (0–1)		0.65 ± 0.12	0.72 ± 0.15	0.2255
Post-operation				
NDI (0–100)		13.6 ± 10.8	20.0 ± 14.0	0.1629
NRS; neck (0–10)		1.6 ± 2.3	2.3 ± 2.7	0.5198
NRS; upper back (0–10)		1.4 ± 2.1	2.0 ± 2.4	0.4517
NRS; arm (0–10)		1.7 ± 2.3	3.5 ± 3.5	0.0424*
EQ-5D (0–1)		0.82 ± 0.14	0.77 ± 0.16	0.3239

Data are shown as mean ± SD or number of participants (%). *: *P* < 0.05. BMI, body mass index; NDI, Neck disability index; NRS, numerical rating scale; EQ-5D, EuroQOL-5D

**Table 6:** Factors related to willingness to undergo the same operation if needed

		Willing (*n* = 36)	Unwilling (*n* = 6)	*P*
Age		53.4 ± 11.2	49.5 ± 16.1	0.4594
Sex	Male (*n* = 34)	82.4	17.6	0.5757
	Female (*n* = 8)	100	0	
BMI (kg/m^2^)		24.7 ± 4.2	21.1 ± 3.0	0.0542
Smoking status	Yes	66.7	33.3	0.2693
	No	87.0	13.0	
Number of operated foramen	1 (*n* = 29)	86.2	13.8	1.0000
	2 (*n* = 13)	84.6	15.4	
Operated side	Right (*n* = 18)	83.3	16.7	1.0000
	Left (*n* = 24)	87.5	12.5	
Pre-operation				
NDI (0–100)		34.8 ± 14.0	15.6 ± 6.0	0.0109*
NRS; neck (0–10)		4.5 ± 3.2	3.7 ± 3.4	0.5575
NRS; upper back (0–10)		4.5 ± 3.5	3.0 ± 3.5	0.3335
NRS; arm (0–10)		5.9 ± 3.2	2.8 ± 3.5	0.0379*
EQ-5D (0–1)		0.65 ± 0.12	0.78 ± 0.11	0.0140*
Post-operation				
NDI (0–100)		14.2 ± 10.6	18.0 ± 17.6	0.4744
NRS; neck (0–10)		1.7 ± 2.3	2.2 ± 2.6	0.6537
NRS; upper back (0–10)		1.4 ± 2.1	2.0 ± 2.5	0.5269
NRS; arm (0–10)		2.1 ± 2.7	1.8 ± 2.7	0.8336
EQ-5D (0–1)		0.80 ± 0.15	0.85 ± 0.11	0.4436

Data are shown as mean ± SD or number of participants (%). *: *P* < 0.05. BMI, body mass index; NDI, Neck disability index; NRS, numerical rating scale; EQ-5D, EuroQOL-5D

## Discussion

In this study, all of the outcome measurement scores for pain and disability improved significantly after the operation, and a large portion of the subjects were subjectively satisfied with the operation. Parker et al. have reported the minimal clinically important difference (MCID) of the NDI, NRS, and EQ-5D for ACDF for CR [17]. They reported that the MCIDs of the NDI, NRS for the neck, NRS for the arm, and EQ-5D are 17.3, 2.6, 4.1, and 0.24, respectively. The differences in the scores in the current study, using the same questionnaires, were 18.0, 2.6, 3.4, and 0.14, respectively. The differences for the NDI and NRS for the neck are equivalent to the previous study; therefore, we can infer that microendoscopic foraminotomy for CR reduced disability and neck pain in a manner similar to ACDF for CR.

We also evaluated whether patients would be willing to undergo the same operation in the future, if needed, as well as subjective satisfaction in order to assess satisfaction both indirectly and directly. The two questions were significantly related; however, some of the results differed between the two questions.

Younger age, non-smoking status, high preoperative NDI score, and low postoperative NRS score for the arm were observed to be significantly different in those who were subjectively satisfied. A systematic review showed that the effectiveness of cervical and lumbar surgery in smokers is lower than that in non-smokers [18]. Our results were consistent with these findings.

The preoperative NDI score of 22.2 in the unsatisfied group was significantly lower than that of 35.4 in the satisfied group. A previous study established that the cut-off value for disability using the NDI is 15 [19]. Although the NDI of both groups indicates the presence of disability, those who were in the unsatisfied group were less disabled preoperatively and therefore had less subjective improvement in pain and disability than expected. Similarly, the difference between the respective preoperative NDI scores of 15.6 and 34.8 in those unwilling and willing to undergo the same operation if needed could be explained by the same aforementioned reason. Although there were no statistically significant differences in the postoperative NDI scores between the satisfied group and the unsatisfied group (13.6 vs. 20.0, respectively), and those willing and unwilling to undergo the same surgery if needed (14.2 vs. 18.0, respectively), only the mean postoperative NDI scores of the satisfied group and those willing to undergo future surgery were less than the disability cut-off value of 15 [19]. In that sense, postoperative disability could also be related to subjective satisfaction. A previous study showed that anterior cervical surgery for CR is regarded as effective when the postoperative NDI is seven or less [20]. The relatively high score of the postoperative NDI in the current study could be attributed to the fact that the posterior approach only provided indirect decompression of the nerve root, which was compressed on the anterior side. A previous study concluded that the NDI is the most valid and responsive measure of the improvement in pain and disability after cervical spine surgery [21], and our results were consistent with this report, confirming that preoperative and postoperative disability could be related to subjective satisfaction.

The mean postoperative NRS score for the arm was 1.7 in the satisfied group, which was significantly lower than that of 3.5 in the unsatisfied group. The mean preoperative NRS score for the arm was 5.9 in those willing to undergo the same operation if needed, which was significantly higher than that of 2.8 in those unwilling to undergo the same operation. Those who were unwilling to undergo the same operation had less arm pain preoperatively and therefore experienced less subjective improvement in pain than expected. Therefore, improvement in arm pain could also be a factor related to subjective satisfaction, as in the previous study [21].

A systematic review of patient-reported scores for quality-of-life outcomes for spine surgery showed that the EQ-5D is a useful tool [22]. The mean preoperative EQ-5D of 0.65 in those willing to undergo the same operation if needed was significantly lower than that of 0.78 in those unwilling to undergo the same operation. As with the NDI and NRS score for the arm, a score of 0.78 might indicate less disability in those unwilling to undergo the same operation if needed.

There were some limitations to the current study. First, we did not compare patient satisfaction with surgical outcomes from other methods of operation, such as ACDF, for CR in this study. Second, we could not perform multivariate analyses because the number of patients who were unsatisfied with the operation and those unwilling to undergo the same operation if needed were too low. Third, the follow-up period was relatively short at 1 year. If we followed these patients for a longer period, the outcomes might have been different. Fourth, there was selection bias among our patients. We were not able to acquire information about the number of those who responded to conservative treatment for CR in our hospital. Since therapy for CR is usually conservative, one indication for the operation is determined by the willingness of the patient to proceed with surgery. As such, the subjects of the current study might have a tendency to be willing to undergo surgery. Furthermore, the patients from one hospital may not represent all patients with CR in general.

## Conclusions

Factors related to subjective satisfaction following microendoscopic foraminotomy for CR include younger age, non-smoking status, high preoperative NDI score, high preoperative NRS score for the arm, low preoperative EQ-5D score, and a low postoperative NRS score for the arm.

## Abbreviations

ACDF: Anterior cervical decompression and fusion
BMI: Body mass index
CR: Cervical radiculopathy
EQ-5D: EuroQOL-5D
MCID: Minimal clinically important difference
NDI: Neck disability index
NRS: numerical rating scale
